# Acute Sciatic Neuritis following Lumbar Laminectomy

**DOI:** 10.1155/2014/404386

**Published:** 2014-06-15

**Authors:** Foad Elahi, Patrick Hitchon, Chandan G. Reddy

**Affiliations:** ^1^Center of Pain Medicine, University of Iowa, 200 Hawkins Drive, 5JPP, Iowa City, IA 52242, USA; ^2^Department of Neurosurgery, University of Iowa, Iowa City, IA, USA

## Abstract

It is commonly accepted that the common cause of acute/chronic pain in the distribution of the lumbosacral nerve roots is the herniation of a lumbar intervertebral disc, unless proven otherwise. The surgical treatment of lumbar disc herniation is successful in radicular pain and prevents or limits neurological damage in the majority of patients. Recurrence of sciatica after a successful disc surgery can be due to many possible etiologies. In the clinical setting we believe that the term sciatica might be associated with inflammation. We report a case of acute sciatic neuritis presented with significant persistent pain shortly after a successful disc surgery. The patient is a 59-year-old female with complaint of newly onset sciatica after complete pain resolution following a successful lumbar laminectomy for acute disc extrusion. In order to manage the patient's newly onset pain, the patient had multiple pain management visits which provided minimum relief. Persistent sciatica and consistent physical examination findings urged us to perform a pelvic MRI to visualize suspected pathology, which revealed right side sciatic neuritis. She responded to the electrical neuromodulation. Review of the literature on sciatic neuritis shows this is the first case report of sciatic neuritis subsequent to lumbar laminectomy.

## 1. Introduction

The sciatic nerve arises from the lumbosacral plexus. The sciatic nerve derives its nerve fibers from the L4, L5, S1, S2, and S3 nerve roots [1].

Sciatica is a sign that is frequently encountered in a clinical practice. Management often poses a problem to clinicians especially if the reason for the pain cannot be identified with great certainty. The independent point prevalence of sciatica in the adult population is greater than 5% and its lifetime prevalence is as high as 40% [2].

The concept of sciatica remains unclear and imprecise, mixing true radicular pain with ordinary lower limb radiating pain. Moreover, even if numerous recommendations are available for the clinical diagnosis and management of lower back pain, the treatment of sciatica and sciatic pain remains poorly defined despite its relatively high prevalence [3].

New diagnostic technology is now able to demonstrate that many cases of sciatica are in fact due to causes unrelated to disc lesions such as piriformis syndrome and distal foraminal impingements. Sciatic neuropathy can be the result of any focal lesion of the nerve in the hip or thigh, distal to the lumbosacral plexus but proximal to the separation of the nerve into its distal branches.

The straight leg rising (SLR) test is commonly positive in a dermatomal distribution among recently diagnosed sciatica. However, the SLR test was shown to be negative in chronic low back pain with leg pain cases. The radicular pain in chronic sciatica is frequently considered dynatomal instead of the dermatomal distribution, and therefore the clinician usually cannot rely on dermatomal pain distribution per se.

Some cases of sciatica, especially secondary to nerve root compression, can present with radiculopathy without radicular pain. Clinical presentation of distal motor, sensory involvement and reflex changes can be useful to identify the exact nerve root involvement. The first step toward diagnosis of the cause of sciatica is thorough past medical history followed by the details of physical examination. Electrodiagnostic study is an extension of physical examination and will play a major role to identify the nerve root(s) involved.

The diagnostic values of electrophysiological tests were evaluated in the literature for sciatica. The electrophysiological study includes dermatomal somatosensory evoked potentials, electromyography, F-wave latencies, H-reflexes, and motor and sensory nerve conduction determinations [4].

Spinal intervention techniques along with peripheral nerve blocks can play a diagnostic and therapeutic role. Interventional pain physicians can play a significant role in identifying the “pain generator.” In the world of pain interventional practice, cases in which the response to an injection (nerve block) is also consistent with the diagnostic criteria will be considered to have “confirmed” the diagnosis.

Even though lumbar myelograms and myelo-CT scans have great value for postsurgical cases, high tesla MRI imaging, magnetic resonance neurography, and interventional MR imaging provide greatly enhanced diagnostic capability for the evaluation of entrapment of the proximal sciatic nerve and its precedent neural elements.

## 2. Case Report

A 59-year-old female initially presented to the neurosurgery clinic with complaints of insidious onset of right leg radicular pain. She has no history of numbness, tingling, weakness, or bowel or bladder incontinency. She has never experienced any similar pain episodes. Physical examination had significant right side straight leg test positive at 45 degrees and left lower extremity at 70 degrees. Plain lumbosacral X-ray was inconclusive. The patient's lumbar MRI reported right L4-L5 lateral disc herniation with extrusions and the patient had partial response to conservative management.

She underwent a minimally invasive hemilaminotomy of right side L4-L5 and removal of the extruded disc particles. The patient had an uneventful postoperative course. She had immediate pain relief after surgery and was able to ambulate on postoperative day one. She was discharged from the hospital the following day after surgery.

The patient reported a new pain experience on the left buttock that started insidiously two weeks after discharge. The pain was constant burning at the left pelvic region, with sharp shooting pain in the left leg. The patient reported pain radiating in multiple trajectories but on clinical assessment had no specific dermatomal distribution. Pain was not aggravated by coughing or sneezing but is more during a sitting position.

On a new physical examination, a healed surgical scar of posterior approach was seen over the midline lower lumbar area. There was no sign of infection at the surgical scar. The patient walked with an antalgic and waddling gait. There was a significant Tinel sign on the left gluteal region over sciatic nerve. Straight leg rising test was negative in supine, sitting, and prone position. Detailed physical examination included spine mobility with great range of motion with no pain, manual muscle testing with normal muscle strength, and deep tendon reflexes were normal. Piriformis stretch test was significantly positive and could aggravate the patient's pain.

The patient had an MRI of lumbosacral, which showed postoperative changes with no disc herniations at the lower lumbar spine.

Throughout the patient's multiple pain clinic visits, we began a multimodality treatment, which included antiepileptic and antidepressant antalgic medication management, muscle relaxant, short acting narcotic medication, and physical therapy.

We decided to perform a diagnostic piriformis muscle block, when the combination of muscle relaxants, antidepressants, and antiepileptic medications did not provide significant relief.

The patient had a piriformis muscle injection under ultrasound guidance where 5 cc of 1% lidocaine solution was injected in the targeted muscle. The patient demonstrated immediate pain relief.

**Figure 1:**
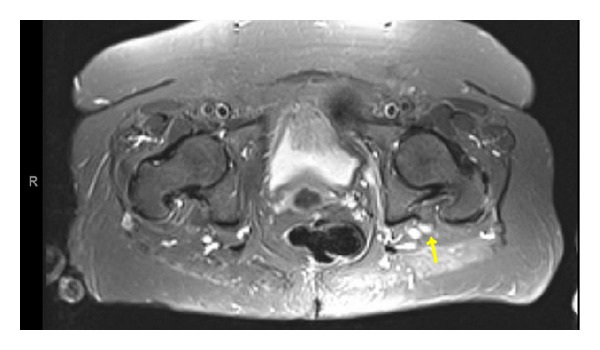
Axial MRI-STIR of the pelvis without contrast. There is asymmetric high T2 signal and enlargement of the left sciatic nerve as seen on axial STIR image localized to pelvic region (marked by yellow arrow).

Repeated piriformis muscle injection was able to provide short course pain relief. We decided to do a pelvic MRI to visualize suspected pathologies. An MRI of the pelvis, without contrast, including coronal T1, STIR, axial T1, T2 fat-sat, and STIR sequences, was done. MRI imaging revealed left side sciatic neuritis (see Figure 1).

With the diagnosis of sciatic neuritis at the left pelvic region, we continued on our multidisciplinary aggressive pain management with a reasonable time frame. The patient was very motivated and followed all steps of the recommendations with significant efforts. She did not respond to the treatment plan after four months of continued aggressive management. We decided to proceed to neuromodulation modality by placing a temporary percutaneous thoracic spinal cord stimulator (SCS). The patient reported a complete response with almost 100% pain relief during one week of the SCS trial. We were able to decrease the patient's medications dramatically during the trial period. The patient then underwent a permanent implant of SCS. On a one-year followup, the patient had remained pain-free without using medication.

## 3. Discussion

Postlumbar spine surgery complication has a wide range, including nerve root injuries, postoperative infection, and steep learning curves for certain procedures.

The type of complication may vary depending on the type of surgical procedure or patient characteristics such as age, gender, comorbidity, smoking, prior surgery, disc levels, and hospital category.

Evidence of nerve root irritation typically manifests as sciatica, a sharp or burning pain radiating down the posterior or lateral aspect of the leg, usually to the foot or ankle. It is well accepted that pain radiating below the knee is more likely to represent true sciatica rather than proximal leg pain [4]. Sciatica pain, due to disc herniation, usually increases with coughing, sneezing, or performance of the valsalva maneuver. In chronic radicular pain or chronic sciatica, pain presents in a dynatomal distribution, which may resemble the distribution of classic dermatomal maps for nerve roots but is not infrequently provoked outside of the distribution of these classic dermatomal maps.

Ryan and Taylor by examining samples of cerebrospinal fluid during administration of intrathecal and epidural injections observed that inflammation was a critical component of radicular pain [5].

Results of postoperative imaging combined with detail history and physical examinations are extremely crucial in order to be able to make decisions for diagnostic spinal injections or piriformis muscle blocks.

According to our knowledge, this is the first case report in the medical literature about postlaminectomy sciatic neuritis. The importance of this case resides in the fact that sciatic neuritis occurs on the left side and the patient had surgery due to right side disc herniation.

The patient was not responsive to multimodality approach for pain management; therefore we chose to perform a spinal cord stimulation implant. Neuromodulation in selected cases either by peripheral or a central nerve stimulation implant will play a major role for neuritis pain management.

We are not able to provide the possible reason(s) for the occurrence of neuritis or to provide a treatment strategy based on this case report. We may consider sciatic neuritis as a rare complication following lumbar laminectomy. Sciatic neuritis should be considered among the differential diagnosis if patients have pain and or deficits within a reasonable gap from surgical intervention.

## 4. Conclusion

It may not be possible to define a precise cause of postoperative sciatica for some patients; however it is important to evaluate and rule out important pathologies and always stay cognizant to these three concerns in taking a history. Is there evidence of systemic disease? Is there evidence of neurologic compromise? Is there social or psychological distress that may contribute to chronic, disabling pain?

If physical examination and medical history are consistent with sciatica and lumbar spine imaging fails to identify a disc herniation consistent with clinical findings then we should thoroughly investigate for other potential causes including sciatic in the pelvic region.
